# Design and simulation of highly efficient CZTS/CZTSSe based thin-film solar cell

**DOI:** 10.1016/j.heliyon.2024.e39903

**Published:** 2024-10-29

**Authors:** Nabila Jahan, Riasat Khan, Mohammad Abdul Matin

**Affiliations:** North South University, Dhaka, 1229, Bangladesh

**Keywords:** Thin-film solar cell, CZTS/CZTSSe, Absorbers, Conversion efficiency

## Abstract

Thin-film solar cells are a substitute for more common crystalline silicon solar cells, which consist of thin semiconductor layers. Thin-film materials comprise direct bandgap and can absorb sunlight more efficiently than silicon. In this article, a double-absorber-based thin-film solar cell comprising CZTS/CZTSSe is designed and optimized through numerical simulation. The proposed solar cell structure consists of a transparent window layer made of aluminum-doped zinc oxide, followed by an intrinsic zinc oxide layer, an n-type cadmium sulfide layer, and a p-type combined absorber layer of copper zinc tin sulfide (Cu_2_ZnSnS_4_) (CZTS) and copper zinc tin sulfur-selenium alloy (Cu_2_ZnSn(S,Se_4_)) (CZTSSe). The structure is further optimized by introducing two interfacial layers between CZTSSe/CZTS and CZTS/CdS. The highest conversion efficiency is achieved by adjusting the thicknesses of the layers, the doping densities in different layers, and the defect densities in the two absorber layers. The optimized model, with a total thickness of 2.01 μm, demonstrates an open-circuit voltage (V${}_{oc}$) of 0.7669 V, a short-circuit current (J${}_{sc}$) of 48.57740 mA/cm^2^, a fill factor (*FF*) of 70.61%, and efficiency (*η*) of 26.31%. These results suggest that CZTS is a promising candidate for replacing other thin-film photovoltaic materials, such as CdTe. The proposed double-absorber thin-film solar cell optimizes doping concentration, thickness, and defect density to enhance performance metrics and efficiency while utilizing non-toxic materials to promote cost-effective, environmentally friendly energy solutions.

## 1 Introduction

Researchers' interest in thin-film photovoltaic cell technology has surged due to the growing demand for inexhaustible and sustainable energy sources. The primary advantage of thin-film solar cells is the reduced material required for their construction. These cells serve as a substitute for the more common crystalline silicon solar cells, consisting of thin semiconductor layers [1]. Thin-film materials have a direct bandgap, allowing them to absorb sunlight more efficiently than silicon, even with much thinner layers. Various materials, such as copper indium gallium selenide (CIGS), cadmium telluride (CdTe), and amorphous silicon, can be used to form thin-film cells. These materials can be mounted on flexible substrates, including plastics or metal foils, thereby requiring less material, reducing costs, and enabling their integration into building-integrated photovoltaics (BIPV) and consumer electronics. While thin-film solar cells generally exhibit lower efficiencies (typically 10-15%) than silicon cells, they perform well in low-light conditions and at high temperatures. Although thin-film cells are less efficient than first-generation solar cells, they offer reduced production costs due to their lower energy and temperature requirements. To date, second-generation thin-film solar cells, including copper indium gallium selenide and cadmium telluride solar cells, have achieved conversion efficiencies of 23.6% and 22.3%, respectively [1]. However, the toxic nature of Cd, the scarcity of Ga in the Earth's crust, and other factors limit the efficiency and increase the cost of these devices. Cd is both toxic and harmful to the environment [2], which has prompted significant research into improving the efficiency of the most well-established solar technologies [3].

Advanced solar cells constructed with the CZTS compound have significantly improved performance since the first recorded device, which had a power conversion efficiency of 0.66% [4]. Thin-film solar cells based on $Cu_{2}ZnSn{\left(S_{x}Se_{1-x}\right)}_{4}$ (CZTSSe) present another viable option because of the plentiful availability of its constituent elements, their nontoxicity, and favorable physical properties. CZTSSe is a strong competitor to existing thin-film solar cell materials due to its high absorption coefficient (exceeding 10^4^ m^−1^) and appropriate direct bandgap (Eg). However, the efficiency of CZTSSe-based thin-film solar cells remains significantly lower than the Shockley-Queisser limit, necessitating further development to meet the demands of the global photovoltaic (PV) market [5].

The Shockley-Queisser limit represents the theoretical maximum efficiency of approximately 33% for a single p-n junction solar cell with a bandgap of 1.4 eV under standard AM1 conditions. Material characteristics significantly impact high-energy conversion devices such as solar cells. Electrical properties, including dielectric constant, doping type (n/p), bandgap energy, and layer thickness, all influence the efficiency of a solar cell. Consequently, researchers are working to improve material quality by modifying the material's composition. Precise material preparation can enhance solar cell efficiency up to the Shockley-Queisser limit. Solar cells represent the most viable option to address the world's increasing energy demands without adversely affecting the environment [6].

Optimizing the structure is critical for achieving higher efficiencies in thin-film solar cells. For instance, the open-circuit voltage ($V_{OC}$) shortage of devices could be substantially minimized, potentially increasing $V_{OC}$ by approximately 15 mV, by replacing conventional CdS buffer layers with alternative buffer layers [7]. A combined absorber layer has been proposed to improve performance. However, CZTSSe is an inherently complex material to handle due to its limited thermodynamic stability, making it challenging to produce a phase-pure substance. The quaternary compound CZTSSe comprises various elements—copper, zinc, tin, sulfur, and selenium—whose interactions complicate the formation of a phase-pure material. Minor variations in composition or synthesis conditions can lead to impurities, undesired phases, or defects, negatively impacting the material's performance in solar cells. Additionally, Sn-compounds are flammable, and CZTS degrades at high temperatures. Several secondary phases, such as $Cu_{2}SnS_{3}$ and ZnS, may coexist if the reaction conditions are not carefully controlled.

The CZTS material exhibits favorable optical and electronic properties comparable to CIGS's, positioning it as a promising candidate for use as an absorber layer in thin-film solar cells. In contrast to CIGS and other thin-film materials such as CdTe, CZTS consists entirely of earth-abundant and non-toxic elements. This study aims to design an ultra-thin cell structure capable of enhancing the efficiency of CZTS and CZTSSe combination-based thin-film solar cells. Despite the complexity of CZTSSe manufacturing, the proposed structure achieves a balance between material thickness and efficiency, resulting in a promising compromise.

This work proposes the design and optimization of a thin-film solar cell with a dual absorber structure constructed with CZTS and CZTSSe compounds. The major contributions of this work are as follows:

• This study proposes a double-absorber-based thin-film solar cell and investigates the impact of absorber layer (CZTS/CZTSSe) doping concentration, defect density, and thickness on multiple performance metrics, i.e., efficiency, fill factor (*FF*), open-circuit voltage ($V_{OC}$), and short-circuit current density ($J_{SC}$). These assessments provide insights into how various material properties influence the overall performance of the solar cell.
• The study utilizes the optimal combination and configuration of the novel Al-ZnO/i-ZnO/CdS/CZTS/CZTSSe layer design to achieve outstanding efficiency.
• Calibrating the doping concentration, thickness, and defect density to configure a well-structured cell design accomplishes optimized performance metrics, including improved short-circuit current density.
• The proposed cell structure demonstrates high efficiency while maintaining an ultra-thin profile of 2.01 μm, reducing costs and improving cost-effectiveness.
• The cell structure uses non-toxic materials, contributing to the advancement of environmentally friendly, green energy technologies.

## 2 Design methodology

The study used a numerical analysis approach using the software SCAPS-1D [10]. The simulation tool adds specific flaws near the mid-gap for further compensation in each layer. The Shockley-Read-Hall model is used for bulk defects, and an extension is used for interface defects. SCAPS can manage constant state lighting and monochromatic or uniformly spectral light. The wavelength area can be restricted to mimic using long and short pass techniques. A “small signal” light can be inserted above the specific region to mimic a spectral response measurement. An exponential absorption rule with a few user factors is considered for all semiconductor layers [11].

The series ($R_{s}$) and shunt resistance ($R_{sh}$) values are considered as $1\times 10^{30}$ and 0 Ω.cm^2^ respectively, in SCAPS-1D, the working temperature is fixed at 300 K. The front contact is where the 800 nm spectral lighting is adjusted. Accordingly, and as needed, different material characteristics are defined for each layer. In this study, the defect density, doping concentration, and layer thicknesses are varied to maximize the efficiency of a double absorber-based solar cell. The SCAPS-1D simulation software's working settings are first established. Material characteristics are also determined at each stratum. We changed the thickness of each layer, including the window TCO layer, the intrinsic layer, the buffer layer, and the two absorption layers, to determine how it affected effectiveness. The doping concentration of each layer is also varied to observe the effects of the parameters fill factor, efficiency, open voltage circuit, and short circuit current. The defect density is primarily varied for the two absorber layers to determine the effect at the cell level. To obtain an optimized PV, the optimum density/concentration of the defect, doping density, and absorber layer thickness are adjusted appropriately.

### 2.1 Material parameters and design structure

In this proposed design, a simple CZTS-based structure is initially considered. Using one-dimensional simulation software, a numerical analysis is performed on the CZTS/CdS structure. A conventional ZnO/CdS/CZTS structure is considered, with some parameters adopted from [12] and reproduced for better contrast. The parameters used in the simulation are presented in Table 1, along with a comparison of the performance between the reference and reproduced values. The output results compare three configurations: Reference, Reproduced with Defect, and Proposed, with the proposed design showing a substantial increase in $J_{SC}$ (48.58 mA/cm^2^) and efficiency (26.31%) compared to both the reference and defect-included scenarios. Despite similar fill factors across the configurations, the proposed structure demonstrates notable improvements in performance metrics such as $V_{OC}$, $J_{SC}$, and overall efficiency. Molybdenum is used as the back contact for this structure. A p-type CZTS absorber layer, n-type CdS, and n-type ZnO-i are used as window layers, with FTO serving as the front contact of the cell structure. A defect density is introduced in the CZTS absorber layer.

**Table 1:** Electrical properties of different layers of the cell ZnO/CdS/CZTS and output results.

	Layers						
		i-ZnO			CdS		CZTS
Thickness (μm)		0.02			0.01		1
Bandgap Energy Eg (eV)		3.37			2.42		1.5
Electron Affinity (eV)		4.6			4.5		4.3
Dielectric Ratio		9			9		10
N-type doping Nd (cm^−3^)		1x1016			3x1016		0
P-type doping Na (cm^−3^)		0			0		3x1016
	Output Results						
	$V_{OC}$ (V)		*J*_*SC*_ (mA/cm^2^)	Fill factor (%)		Eff (%)	
Reference	0.44		25.56	75.93		8.38	
Reproduced with defect	0.74		18.24	60.36			
						8.14	
Proposed	0.77		48.58	70.61		26.31	

It is observed that the parameters $J_{SC}$, fill factor, and efficiency are all lower than those in the reference. The $V_{OC}$ is found to be 0.7394 V, $J_{SC}$ is 18.244 mA/cm^2^, the fill factor is approximately 60.36%, and the overall efficiency is 8.14%. The defect density is set at $1\times 10^{17}$. Defects at the absorber/buffer interface and band deviation significantly impact the $V_{OC}$ values. Impurities, crystallographic defects, and interface defects can enhance charge carrier recombination, lowering both $J_{SC}$ and efficiency. The results obtained in this work correspond closely with the corresponding parameters reported in [12].

To further optimize the proposed structure, a back surface field-based layer constructed from a double absorber CZTSSe and an Aluminum doped window layers are employed. The rear surface of solar cells has an integrated field created usually using highly doped and relatively low bandgap materials. The p+/p semiconductor junction develops a built-in field when a p+ semiconductor lies between the p-absorber and back contact.

**Figure 1:**
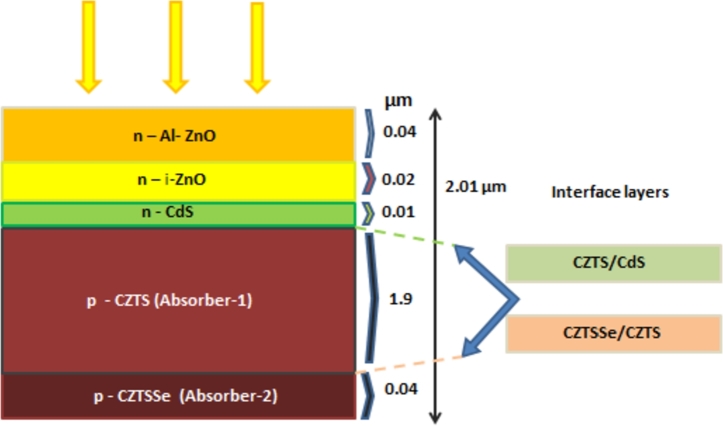
Proposed device structure of Al-ZnO/i-ZnO/CdS/CZTS/CZTSSe.

**Figure 2:**
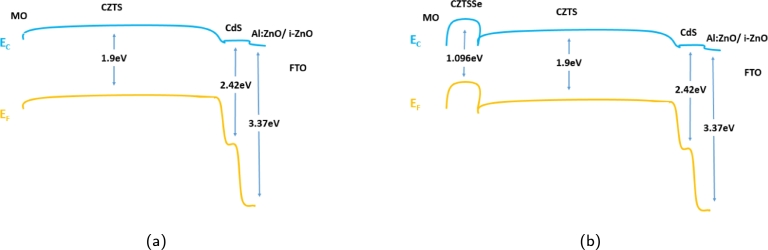
Energy bandgap diagram of *CZTS* solar cell structure (a) without *CZTSSe* (b) with *CZTSSe*.

The proposed design of the cell structure has been presented and schematized in Fig. 1, which shows the structure comprising the material layers Al-ZnO/i-ZnO/CdS/CZTS/CZTSSe. The structure consists of five material layers, each having designated roles and effects on the cell structure. The topmost layer is n-type, the window layer composed of Al-ZnO and intrinsic i-ZnO, with a doping concentration of $3\times 10^{19}$ cm^−3^ and $1\times 10^{16}$ cm^−3^ respectively. Following that, an n-type buffer layer of cadmium sulfide has been used with a doping concentration of $3\times 10^{16}$ cm^−3^. Finally, a p-type double absorber layers of copper zinc tin sulfide $Cu_{2}ZnSnS_{4}$ (CZTS) and sulfur-selenium alloy $Cu_{2}ZnSn{\left(S,Se\right)}_{4}$ (CZTSSe) of bandgap 1.5 eV and 1.096 eV with doping of $3.5\times 10^{16}$ cm^−3^ and $1\times 10^{16}$ cm^−3^ respectively, has been used. We have used $Cu_{2}ZnSnS_{4}$ (CZTS) and sulfur-selenium alloy $Cu_{2}ZnSn{\left(S,Se\right)}_{4}$ (CZTSSe) as absorber layers, which are deposited on Mo-coated glass substrates. It is an essential component in solar cell construction as it provides the necessary physical support for a thin film and affects the structure's overall efficiency. Mo-coated glass substrates have high electrical conductivity, allowing charge transfer and collection. Due to its high melting point and good thermal conductivity, it helps maintain structural veracity. The Mo layers serve as a diffusion barrier, and the coating enhances the adhesion of the absorber layers to the glass substrate. Influencing factors like thermal conductivity, defect density, and charge carrier recombination rates affect conversion efficiency. The interactions of the substrate with the absorber layers affect the defect density, which then impacts the efficiency of the cell. The CZTSSe essentially acts as a buffer layer to improve the efficiency of the structure. The overall thickness of the device structure has been optimized to be the value of 2.01 μm tailoring the device structure to very thin and light. The current CdTe-based cell thickness can be seen around 4-6 μm, and CIS (or CIGS) based solar cells are approximately 3–4 μm, as seen from the literature review. Hence, the proposed design suggests a cell model with a very thin thickness. Energy bandgap diagrams of the structure without and with the addition of CZTSSe have been shown in Fig. 2(a) and Fig. 2(b), respectively. Al: ZnO is the front electrical contact while remaining transparent to incident solar energy and undetectable to visible light. Making a hetero-structure p-n junction with a p-type CZTS topmost absorption layer and ZnO layer function as the window layer and n-type CdS layer serves as the buffer layer [13]. Al-ZnO is utilized for its affordability, nontoxicity, and relatively low cost. In solar cell applications, the current flow in an electrical channel (a parallel adoption from top to bottom layer) must be halted. Since intrinsic ZnO (i-ZnO) is a sheet, this research limits the thickness to less than 50 nm. The aluminum-doped ZnO layer has been improved by altering the thickness. The buffer layer is one of the critical components influencing a solar cell's photovoltaic properties and is essential to its formation. From a physics perspective, the buffer layer affects the overall band structure and provides band integration between the absorber and window deposits. Consequently, the window layer's effect on interfacial stress and defects is reduced. The doping of this layer plays an essential role in the overall structure. The n-type buffer layer has been implemented using CdS. Since this is an n-type material, a buffer layer must have a more significant band gap to guarantee that most light can travel through to the absorber layer [14]. This layer can be improved by changing its thickness and donor concentration to create a better-optimized cell structure, as this study does. The device's performance is improved by the double absorber layer formed by CZTS and CZTSSe working together to increase light absorption by harvesting a broader spectrum of visible light. The differences in short- and long-wavelength performance are linked to the alignment of the bandgap energies in the absorber layers and the device's enhanced optoelectronic properties at the CZTS/CZTSSe interface and the back contact. The series impedance of the double-absorber structure was lower than that of the traditional single-absorber CZTS structure, which resulted in a notable increase in I-V characteristics [14]. Two interface layers of concentration $1\times 10^{14}$ cm^2^ and $1\times 10^{13}$ cm^2^ have also been added in between the layers CZTSSe/CZTS and CZTS/CdS. In addition to offering particular links for one type of carrier and the possibility of organic solar technology, interface materials may include quasi, semi-conducting, or conducting layers that can act as optical or protective layers. Table 2, Table 3, Table 4 depict the device's mathematical and physical specifications of all the material layers in the proposed structure.

**Table 2:** Electrical properties of different layers of the cell.

	Layer				
	Al-ZnO	i-ZnO	CdS	CZTS	CZTSSe

Thickness (μm)	0.04	0.02	0.01	1.9	0.04
Bandgap Energy Eg (eV)	3.37 [5]	3.37 [5]	2.42 [8]	1.5 [5]	1.096 [5]
Electron Affinity (eV)	4.6 [2]	4.6 [9]	4.5 [5]	4.3 [5]	4.1 [5]
Dielectric Ratio	9 [2]	9 [9]	9 [2]	10 [9]	13.6 [5]
N-type doping *N*_*d*_ (cm^−3^)	3 × 10^19^	1 × 10^16^	3 × 10^16^	0	0
P-type doping *N*_*a*_ (cm^−3^)	0	0	0	3 × 10^16^	1 × 10^16^

**Table 3:** Interfacial parameters of interface layers of CZSTSSe/CZTS and CZTS/CdS.

	CZSTSSe/CZTS	CZTS/CdS
Defect Type	Neutral	Neutral
Capture Cross section electrons (cm^2^)	1 × 10^19^	1 × 10^19^
Capture Cross section holes (cm^2^)	1 × 10^19^	1 × 10^19^
Energetic Distribution	Single	Single
Energy with respect to Reference (eV)	0.6	0.6
Total Density (1/cm^2^)	1 × 10^14^	1.5 × 10^13^

**Table 4:** Electrical parameters of contacts of the cell Structure.

	Front Contact	Back Contact
Electrons	1 × 10^7^	1 × 10^5^
Holes	1 × 10^5^	1 × 10^7^
Metal Work function (eV)	4.5323 (FTO)	5.0019 (Mo)
Majority Carrier barrier Height (eV)	-0.0677	0.1941
Flat band	✓	✓

## 3 Results and discussion

Upon extensive analysis, the layer thicknesses of the Al-ZnO/i-ZnO/CdS/CZTS/CZTSSe solar cell have been optimized for the most appropriate configuration. A 1 m^2^ solar panel with an efficiency of 75% would convert an incident solar energy of 1000 W/m^2^ into 750 W of usable electrical energy. Solar cell efficiency is a crucial metric determining the fraction of solar energy converted into useful electrical energy. The fill factor (*FF*), defined by the ratio of maximum power output to the product of open-circuit voltage and short-circuit current, can be used to calculate the efficiency of a solar cell.

(1) $$FF=J_{max}V_{max}/J_{SC}V_{OC}$$

(2) $$\eta =J_{max}V_{max}FF/P_{in}$$

In (1) and (2), $P_{in}$ represents the incoming solar radiation (expressed in W/m^2^), respectively. Estimates of the cell layout, shunt protection, and diode losses all directly affect the fill factor. A higher fill factor enhances efficiency by increasing the shunt resistance ($R_{sh}$) and reducing the series resistance ($R_{s}$), bringing the output power of the photovoltaic effect closer to its theoretical maximum. The fill factor is a critical parameter in determining the efficiency of a PV module, with its maximum value depending on various influencing factors.

### 3.1 Effect of thickness and doping concentration of window layers

**Figure 3:**
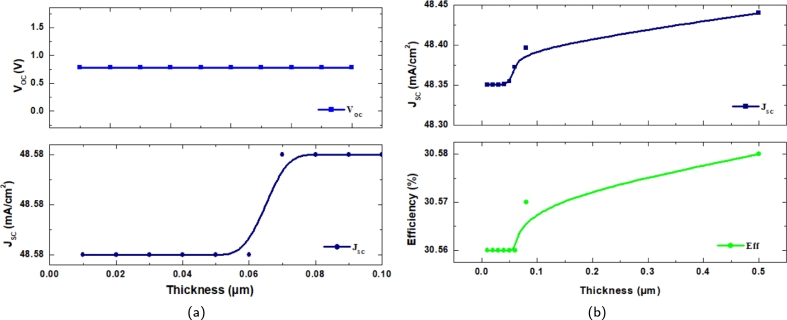
Effect of thickness on *V*_*OC*_, *J*_*SC*_ and *η* of window layer of (a) Al-ZnO (b) i-ZnO.

In the proposed simulation attempted in this work, the thicknesses of Al-ZnO and i-ZnO are varied from 0.01−0.1 μm and 0.01−0.5 μm, respectively. For both window layers, it is found that $V_{OC}$ remains constant across the entire range of thicknesses. The variation in conversion efficiency as a function of Al-ZnO and i-ZnO-based window layer thickness are shown in Fig. 3(a) and Fig. 3(b), respectively. It is evident that as the window layer thickness increases (from 0.01 to 0.5 μm), the conversion efficiency increases asymptotically, following the same trend as $J_{SC}$. The highest conversion efficiency, η=30.64%, is remarkable; however, indiscriminately increasing the thickness would lead to erroneous results.

**Figure 4:**
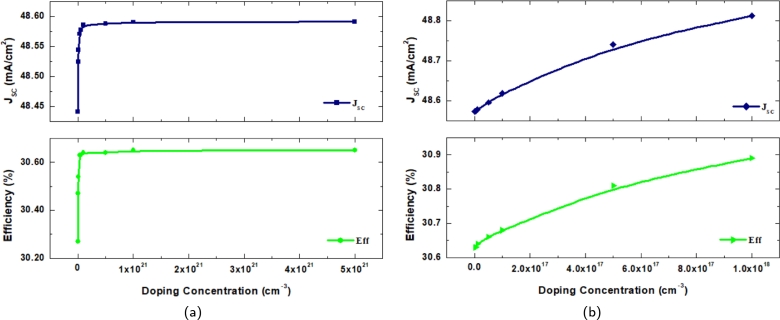
Effect of doping concentration on *J*_*SC*_ and *η* of window layer of (a) Al-ZnO (b) i-ZnO.

The thicknesses of Al-ZnO and i-ZnO are settled to be 0.04 μm and 0.02 μm, which resulted in an efficiency of η=30.64%. With the optimized thicknesses, the doping concentration of the window layers Al-ZnO and i-ZnO are varied over a range of $1\times 10^{18}$ cm^−3^ to $1\times 10^{22}$ cm^−3^ and $1\times 10^{5}$ cm^−3^ to $1\times 10^{18}$ cm^−3^. Doping concentration refers to the intentional introduction of dopants or impurities into the layers of a solar cell to modify their electrical properties. This adjustment alters the carrier concentration of holes or electrons in the layers, which in turn affects key parameters such as $V_{OC}$, $J_{SC}$, fill factor, and overall efficiency. It is observed that with the increase in the doping concentration of the window layers, the $J_{SC}$ and the efficiency gradually increased with the rise of the concentration but at a slow rate. The $V_{OC}$ generally remained constant throughout the simulation with little to no change. Fig. 4(a) and Fig. 4(b) illustrate the effects of doping concentration for Al-ZnO and i-ZnO, respectively. Though there is a gradual increase in both parameters, the values reach a certain saturation after a point. The fill factor shows a similar change as the efficiency.

### 3.2 Effect of thickness and doping concentration of buffer layer

**Figure 5:**
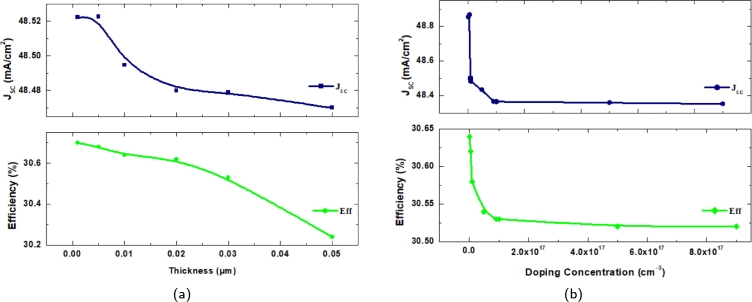
Effect of (a) Thickness and (b) Doping Concentration *J*_*SC*_ and *η* of the proposed solar cell.

The effect of the CdS buffer layer's thickness (measured in the range of 0.001-0.05 μm) on the various photovoltaic characteristics of the suggested solar cell has been demonstrated in this section. Fig. 5(a) and Fig. 5(b) show the impact of thickness and doping concentration on the proposed solar cell's short-circuit current density and efficiency, respectively. It is observed that as the thickness and doping concentration increase, both $J_{SC}$ and efficiency experience a decline, with notable reductions in performance at higher doping levels and thickness values beyond 0.02 μm. This can be explained in terms of the loss of electron-hole pairs produced by photos. The distance that the carriers must journey to the junction is actually increased as the buffer layer thickness rises, and so is the likelihood that they will recombine before reaching the area where the junction's electric potential will separate them. This particular type of loss in solar cells is well documented. The buffer layer thickness change does not impact the open-circuit voltage $V_{OC}$, oscillating around 0.7831 V.

The efficiency decreases gradually with the increase of the thickness of the buffer layer. It can be seen that the optimal range of the thickness is around 0.01-0.02 μm, showing a generally stable value. The greatest conversion efficiency of 30.64% is achieved when the CdS buffer layer is used with the recommended thickness range of 0.0.1- 0.02 μm. $V_{OC}$ again has little to no change and remains relatively constant throughout, and the fill factor behaves in a similar tone in accordance with the efficiency. The $J_{SC}$ values and the efficiency decrease gradually, saturating between concentrations, but overall does decrease. The doping concentration of the layers has been observed through the range of $1\times 10^{15}$ cm^−3^ to $9\times 10^{18}$ cm^−3^. The values of all the parameters are seen to be at the approximate ranges between $1\times 10^{15}$ cm^−3^ to $5\times 10^{16}$ cm^−3^ as seen in Fig. 5.

### 3.3 Effect of thickness and defect density of absorber layer

**Figure 6:**
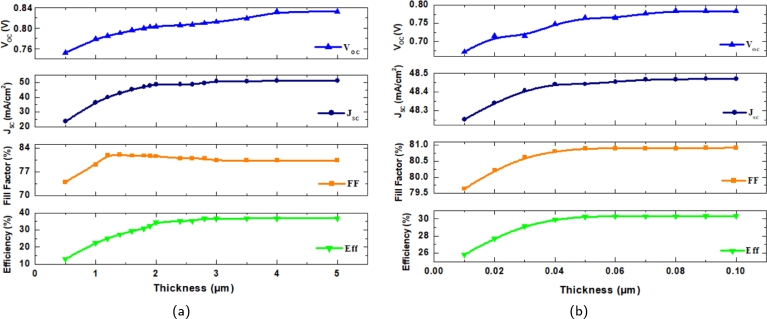
Impact on *V*_*OC*_, *J*_*SC*_ and *η* with thickness of absorber layer (a) CZTS (b) CZTSSe.

The thickness of the absorption layers CZTS and CZTSSe has been explored in the subsequent paragraphs. Selecting the appropriate absorber thickness is crucial for an effective solar cell. Fig. 6(a) shows that as the CZTS thickness increases, the short-circuit current density $J_{SC}$ rises. The CZTS is varied between 0.5 to 5 μm, whereas the second absorber layer CZTSSe has been varied from 0.01 to 0.1 μm. The proposed thin CZTSSe makes the structure thinner and cost-effective. The open-circuit voltage $V_{OC}$, drops as the CZTS thickness rises as expected. The increase of $J_{SC}$ is due to a more significant gathering of incident photons with higher energies. Pure sulfur $Cu_{2}ZnSnS_{4}$ (CZTS) thin-film solar cells' current performance is primarily constrained by low $V_{OC}$. Both fill factor and efficiency are observed to increase initially with the increase of the absorber layer CZTS in Fig. 6(a) and absorber layer CZTSSe in Fig. 6(b), but soon come to a saturation level. As the absorber's thickness changes, the fill factor varies as well. It is observed that the fill factor is elevated initially as the thickness increases. However, the fill factor begins to decrease when the thickness of the absorber layers is raised for CZTS beyond 2μm and for CZTSSe beyond .05μm. The impact of recombination causes this decrease in fill factor with an increased absorption layer. Recombination rises along with the thickness. After a certain point, this influence causes the fill factor to reach saturation and beyond that decline. Both the absorber layers show similar results regarding their thickness increases to improve the solar cell structure.

**Figure 7:**
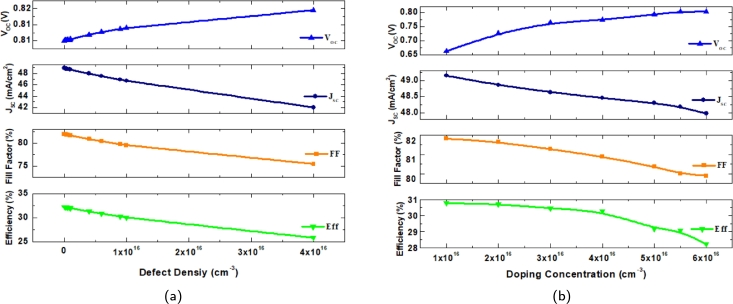
Effect on *V*_*OC*_,*J*_*SC*_, FF and *η* with (a) defect density (b) doping concentration of absorber layers.

Fig. 7(a) depicts the effect of the defect concentrations of the CZTS/CZTSSe substrate. The defect density in CZTS and CZTSSe arises from defects or irregularities within the crystal structure of these semiconductor materials. Defect density significantly affects the electrical properties and efficiency of photovoltaic cells. Higher defect concentrations generally lead to increased recombination rates, where electron-hole pairs recombine before contributing to the electric current. These defects can also hinder carrier mobility and reduce light absorption capacity. For the CZTS, the defect density is varied from $1\times 10^{9}$ cm^−3^ to $4\times 10^{16}$ cm^−3^ and for CZTSSe $1\times 10^{13}$ cm^−3^ to $1\times 10^{18}$ cm^−3^. All four performance measures of the cell structure, such as efficiency, $J_{SC}$, fill factor, and $V_{OC}$, decrease with the increase of defect density. This scenario is due to the elimination of the charge carriers caused by the improvement in the recombination technique, which accounts for the deterioration in the cell's performance with increasing defect density. The carrier diffusion length is considerable, and the recombination rate is low at low defect densities, contributing to improved PV performance. Double absorber layer defects are reduced to the lowest feasible values to increase photovoltaic efficiency.

### 3.4 Effect of the doping concentration of absorber layers

In this section, the doping concentration of the absorber layers has been demonstrated. Both CZTS and CZTSSe are combined together for better cell output. However, increasing absorber layer doping concentration has resulted in better outcomes. The layers were doped and varied from $1\times 10^{16}$ cm^−3^ to $6\times 10^{16}$ cm^−3^ and $1\times 10^{13}$ cm^−3^ to $1\times 10^{18}$ cm^−3^ respectfully. A lower doped buffer layer helps to align energy levels. It can minimize carrier recombination between layers. It can be observed in Fig. 7(b) that the open current voltage is seen to rise with the increase in the doping concentration. However, it reaches saturation after the extending $1\times 10^{18}$ cm^−3^. This increase in the $V_{OC}$ can result from the due to improvement of overload current in the structure. Besides this, the fill factor, $J_{SC}$ and efficiency decrease with the increase of the doping concentration.

### 3.5 Limitations of the proposed system

While the proposed cell structure offers several advantages, it also presents certain drawbacks, as with any design. In CZTS/CZTSSe-based cells, the higher density of defects leads to increased defect-activated recombination, which reduces overall efficiency. Furthermore, controlling defects during large-scale production remains challenging. This design requires the precise control of multiple layers, particularly concerning properties such as thickness and doping, to achieve optimal performance and high efficiency. However, maintaining such precision during manufacturing can be difficult. Additionally, thin-film cells tend to be less stable under heat and other environmental conditions compared to their crystalline counterparts, potentially affecting long-term reliability.

## 4 Optimization of device structure and performance comparison

**Figure 8:**
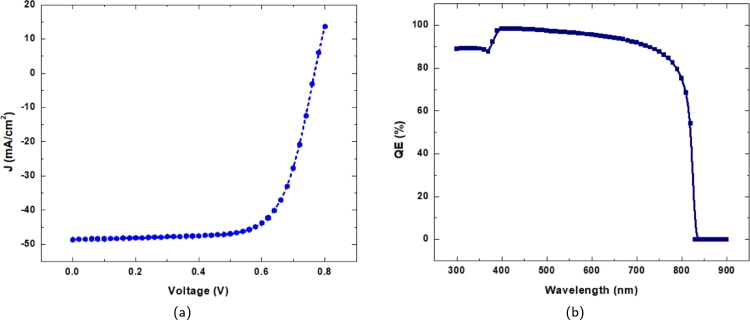
(a)I-V curve (b) QE curve for Al-ZnO/i-ZnO/CdS/CZTS/CZTSSe.

One of the prime focuses of this research is to design a thin cell structure with optimum performance using CZTS and CZTSSe. Two interfacial layers are introduced between CZTSSe/CZTS and CZTS/CdS. Through the systematic simulation study, the parameters of the device structure are chosen, and we can achieve an open circuit voltage of 0.7669 V, short circuit current 48.57740 mA/cm^2^, a fill-factor 70.61% and an efficiency of 26.31%. Two interfacial layers of concentration of $1\times 10^{14}$ cm^2^ and $1.5\times 10^{13}$ cm^2^ are chosen respectively to achieve these results. Fig. 8(a) and Fig. 8(b) display the I-V and QE curves of the optimized result. The I-V curve of a solar cell shows the electrical characteristics of the cell under various conditions of operation. In Fig. 8(a), the slope of the curve at a specific point reflects the electrical resistance of the cell at that particular point. The steeper slope indicates low electrical resistance, which means the cell is more effective at transforming solar energy into electrical energy. The photovoltaic cell's QE (quantum efficiency) curve shows the cell's capacity to transform photon energies into electrical power. Thus, the transformed electrical current is a function of photon energy. The QE curve is fairly squarish and provides improved performance, as illustrated in the figure.

Table 5 presents a comparative analysis of the proposed thin-film solar cell against related works in terms of important photovoltaic parameters, e.g., $V_{OC}$, $J_{SC}$, fill factor, and efficiency. The proposed structure, CZTS/CZTSe/CdS, shows superior efficiency at 26.31% compared to the others, including a notable improvement over the highest $J_{SC}$ (48.5774 mA/cm^2^). Despite having a lower $V_{OC}$ (0.7669 V), the proposed solar cell's overall performance surpasses that of other designs, underscoring the effectiveness of the proposed structure in enhancing solar cell efficiency.

**Table 5:** Comparison of the proposed thin-film solar cell with related works.

Reference	Structure	Photovoltaic Parameter				Study type
		*V*_*OC*_(*v*)	*J*_*SC*_ (mA/cm^2^)	FF(%)	*η* (%)	

[13]	CZTS/CZTSe/CdS	1.324	20.98	78.2	21.7	Simulation
[15]	CZTS/Si/CdS	0.84	27.55	88.54	19.4	Simulation
[16]	CZTS/CdS	1.45	-	-	23.56	Simulation
[17]	CZTSe/CdS	0.5431	45.87	80.55	20.07	Simulation
Proposed	CZTS/CZTSse/CdS	0.7669	48.5774	70.61	26.31	Simulation

## 5 Conclusion

In this article, our goal is to design and optimize a thin cell structure with optimal performance. With the elimination technique, numerical analysis, comparative study, and a thorough simulation study, we develop a very thin structure Al-ZnO/i-ZnO/CdS/CZTS/CZTSSe and two interfacial layers to improve upon the output performance. CZTS is an excellent choice for cell structure because it is affordable and nontoxic. With the proposed structure, we have obtained an open circuit voltage of 0.7669 V, short circuit current 48.57740 mA/cm^2^, a fill-factor 70.61% and an efficiency of 26.31%. In the future, the proposed solar cell's design can be extended to adaptable solar panels, which might be incorporated into various technologies like wearable electronics and portable solar chargers. By replacing potentially hazardous elements in conventional solar cells, the research paves the way for more environmentally friendly production procedures and a decrease in the environmental impact of solar energy technologies. The knowledge gathered from this research may be used to create multi-junction solar cells that can absorb extensible sunlight.

## CRediT authorship contribution statement

**Nabila Jahan:** Writing – original draft, Visualization, Validation, Software, Methodology, Investigation, Data curation, Conceptualization. **Riasat Khan:** Writing – review & editing, Supervision, Resources, Project administration, Formal analysis, Conceptualization. **Mohammad Abdul Matin:** Writing – review & editing, Visualization, Supervision, Project administration, Investigation, Formal analysis, Conceptualization.

## Declaration of Competing Interest

The authors declare that they have no known competing financial interests or personal relationships that could have appeared to influence the work reported in this paper.

## Data Availability

No data was used for the research described in the article.

## References

[1] Elkhamisy K., Abdelhamid H., El-Rabaie E.-S., Abdelsalam N. (2023). A comprehensive survey of silicon thin-film solar cell: challenges and novel trends. Plasmonics.

[2] Tripathi S., Lohia P., Dwivedi D. (2020). Contribution to sustainable and environmental friendly non-toxic CZTS solar cell with an innovative hybrid buffer layer. Sol. Energy.

[3] Khemiri N., Chamekh S., Kanzari M. (2020). Properties of thermally evaporated CZTS thin films and numerical simulation of earth abundant and non toxic CZTS/Zn(S,O) based solar cells. Sol. Energy.

[4] Kumar M.S., Madhusudanan S.P., Batabyal S.K. (2018). Substitution of Zn in earth-abundant $Cu_{2}ZnSn{\left(S/Se\right)}_{4}$ based thin film solar cells–a status review. Sol. Energy Mater. Sol. Cells.

[5] Abderrezek M., Djeghlal M.E. (2021). Numerical study of CZTS/CZTSSe tandem thin film solar cell using SCAPS-1D. Optik.

[6] Pal K., Singh P., Bhaduri A., Thapa K.B. (2019). Current challenges and future prospects for a highly efficient (> 20%) kesterite CZTS solar cell: a review. Sol. Energy Mater. Sol. Cells.

[7] Nakamura M., Yamaguchi K., Kimoto Y., Yasaki Y., Kato T., Sugimoto H. (2019). Cd-free $Cu(In,Ga){\left(Se,S\right)}_{2}$ thin-film solar cell with record efficiency of 23.35%. IEEE J. Photovolt..

[8] Abdelkadir A.A., Oublal E., Sahal M., Gibaud A. (2022). Numerical simulation and optimization of n-Al-ZnO/n-CdS/p-CZTSe/p-NiO (HTL)/Mo solar cell system using SCAPS-1D. Results Opt..

[9] Maurya K., Singh V. (2022). $Sb_{2}Se_{3}/CZTS$ dual absorber layer based solar cell with 36.32% efficiency: a numerical simulation. J. Sci. Adv. Mater. Dev..

[10] Burgelman M., Marlein J. (2008). European Photovoltaic Conference.

[11] Burgelman M., Nollet P., Degrave S. (2000). Modelling polycrystalline semiconductor solar cells. Thin Solid Films.

[12] Toura H., Khattak Y.H., Baig F., Soucase B.M., Touhami M.E. (2019). Back contact effect on electrodeposited CZTS kesterite thin films experimental and numerical investigation. Sol. Energy.

[13] Gupta G.K., Dixit A. (2018). Theoretical studies of single and tandem $Cu_{2}ZnSn{\left(S/Se\right)}_{4}$ junction solar cells for enhanced efficiency. Opt. Mater..

[14] Hayakawa T., Nishimura T., Sugiura H., Suyama N., Nakada K., Yamada A. (2018). Control of donor concentration in n-type buffer layer for high-efficiency $Cu(In,Ga)Se_{2}$ solar cells. IEEE J. Photovolt..

[15] AlZoubi T., Moghrabi A., Moustafa M., Yasin S. (2021). Efficiency boost of CZTS solar cells based on double-absorber architecture: device modeling and analysis. Sol. Energy.

[16] Gueddim A., Bouarissa N., Naas A., Daoudi F., Messikine N. (2018). Characteristics and optimization of ZnO/CdS/CZTS photovoltaic solar cell. Appl. Phys. A.

[17] Mathur A.S., Dubey S., Singh B. (2020). Study of role of different defects on the performance of CZTSe solar cells using SCAPS. Optik.

